# Supplementary material for: The plasma degradome reflects later development of NASH fibrosis after liver transplant
Source: Sci Rep. 2023 Jun 20;13:9965. doi: 10.1038/s41598-023-36867-x (PMC10282030; doi:10.1038/s41598-023-36867-x)
Supplement: Supplementary file 1 — Supplementary Information. [file 41598_2023_36867_MOESM1_ESM.pdf]

SUPPLEMENTAL MATERIAL

The plasma degradome reflects later development of NASH fibrosis after liver transplant

Jiang Li[1], Toshifumi Sato[1], María Hernández-Tejero[1] Juliane I. Beier[1,2], Khaled Sayed[3], Panayiotis V Benos[3], Daniel W Wilkey[4], Abhinav Humar[5], Michael L Merchant[4], Andres Duarte-Rojo[7,8], Gavin E Arteel[1,2]

[1]Department of Medicine, [2]Pittsburgh Liver Research Center, University of Pittsburgh, Pittsburgh, PA, USA.

[3]Department of Epidemiology, University of Florida, Gainesville, FL, USA,

[4]Department of Medicine, University of Louisville, Louisville, KY, USA.

[5]Department of Surgery, University of Pittsburgh, Pittsburgh, PA, USA.

[6]Division of Gastroenterology and Hepatology, [7]Comprehensive Transplant Center, Northwestern Medicine and Feinberg School of Medicine, Northwestern University, Chicago, IL.

Running title: The degradome in post-LT NASH fibrosis.

Key Words: Extracellular matrix, remodeling, liver transplantation outcomes, translational study

Send all correspondence to: Gavin E. Arteel, PhD, FAASLD

Thomas E. Starzl Biomedical Science Tower

West 1143

200 Lothrop Street

Pittsburgh, PA 15213

Phone: +1-412-648-4187

Email: gearteel@pitt.edu

Abbreviations: ALT, alanine aminotransferase; AST, aspartate aminotransferase; BW; body weight; CVD, cardiovascular disease; ECM, extracellular matrix; GO, gene ontology; HFD, high-fat diet; HOMA-IR, homeostatic model assessment for insulin resistance; KEGG, Kyoto encyclopedia of genes and genomes; LC-MS/MS, liquid chromatography, tandem mass-spectroscopy; LFD, low-fat diet; Log2FC, fold-change (log2); LT, liver transplantation; MetS, metabolic syndrome; MMP, matrix metalloproteinases; MS, mass spectrometry; NAFLD, non-alcohol-related fatty liver disease; NASH, non-alcoholic steatohepatitis; OPLS-DA, orthogonal partial least squares discriminant analysis.

Supplemental Methods

Information on rtPCR primers and probes, drugs and chemical assays and antibodies used in this study are summarized in supplemental Tables 1-2.

Biochemical assays and histology. Plasma levels of Aspartate transaminase (AST) and alanine amino transferase (ALT) were determined spectrophotometrically using standard kits (Thermo Fisher Scientific, Waltham, MA), as described previously.[1] Formalin-fixed, paraffin embedded sections were cut at 5 μm and mounted on glass slides. Deparaffinized sections stained with hematoxylin and eosin and pathology was assessed in a blinded manner. ECM accumulation in liver sections was determined by staining with Sirius red/fast green and were visualized via brightfield analysis.[2] Hepatic lipids (NEFA, TG and TC) were determined spectrophotometrically, as described previously.[3] Fasting plasma glucose and insulin levels were determined using commercially-available kits (Jiang need information). HOMA-IR was calculated using the following equation: fasting insulin (ng/mL)×fasting blood glucose (mg/dL)/405.[4]

RNA Isolation and Real-Time RT-PCR RNA extraction and real-time RT-PCR were performed as described previously.[5] RNA was extracted immediately following sacrifice from fresh liver samples using RNA Stat60 (Tel-Test, Ambion, Austin, TX) and chloroform. RNA concentrations were determined spectrophotometrically and 1μg of total RNA was reverse transcribed using the QuantiTect Reverse Transcription Kit (Qiagen,Valencia, CA). Real-time RT-PCR was performed using a StepOne real time PCR system (Thermo Fisher Scientific, Grand Island, NY) using the Taqman Universal PCR Master Mix (Life Technologies, Carlsbad, CA). Primers and probes were ordered as commercially available kits (Thermo Fisher

Scientific, Grand Island, NY). Primer sequences from Taqman Gene Expression Assays (Applied Biosystems, Foster City, CA) were as follows: Collagen type I alpha 1 (Col1α1), Actin Alpha 2, Smooth Muscle (ACTA2), Transforming Growth Factor Beta 1 (TGFB1), Prolyl 4-Hydroxylase, Transmembrane (P4htm), protein-lysine 6-oxidase (LOX1,2), MMP- 2, 8, 9, 12, 13, and 14. The comparative C_T method was used to determine fold differences between the target genes and an endogenous reference gene (18S). Results were reported as copy number $2^{-\Delta CT}$.

Supplemental Results

Supplemental Table 4 summarizes results of cluster analysis of significantly increased peptides by StringDB, which is also shown graphically in Figure 5. Supplemental Table 5 lists all peptides significantly increased in mouse plasma 1D and/or 28D after cessation of CCl_4, as described in Experimental Procedures, which is shown graphically in Figure 5. Supplemental Table 6 summarizes feeding, growth and lipid accumulation caused by HFD in C57Bl6/J and AJ mice (see also Figure 3). Supplemental Figure 1 compares mRNA expression of key genes involved in hepatic inflammation and fibrosis in C57Bl6/J and AJ mice fed HFD or LFD for 12 weeks (see also Figure 3).

Supplemental Reference List

1 Massey, V. L. *et al.* The hepatic "matrisome" responds dynamically to injury: Characterization of transitional changes to the extracellular matrix in mice. *Hepatology* **65**, 969-982 (2017). https://doi.org:10.1002/hep.28918

2 Rittié, L. Method for Picrosirius Red-Polarization Detection of Collagen Fibers in Tissue Sections. *Methods Mol Biol* **1627**, 395-407 (2017). https://doi.org:10.1007/978-1-4939-7113-8_26

3 Tan, M. *et al.* Chronic subhepatotoxic exposure to arsenic enhances hepatic injury caused by high fat diet in mice. *Toxicol Appl Pharmacol* **257**, 356-364 (2011). https://doi.org:10.1016/j.taap.2011.09.019

4 Parks, B. W. *et al.* Genetic architecture of insulin resistance in the mouse. *Cell Metab* **21**, 334-347 (2015). https://doi.org:10.1016/j.cmet.2015.01.002

5 Isaacson, R. H. *et al.* Olanzapine-induced liver injury in mice: aggravation by high-fat diet and protection with sulforaphane. *J Nutr Biochem* **81**, 108399 (2020). https://doi.org:10.1016/j.jnutbio.2020.108399

Supplemental Table 1-Product information for primers used in RT-PCR

Gene name	Supplier	Cat. No.
18s	Thermofisher	Mm03928990_g1
Col1a1	Thermofisher	Mm00801666_g1
Capn1	Thermofisher	Mm00482964_m1
Capn2	Thermofisher	Mm00486669_m1
Mep1a	Thermofisher	Mm00484970_m1
Tnfa	Thermofisher	Mm00443258_m1
Il10	Thermofisher	Mm01288386_m1
Serpine1	Thermofisher	Mm00435858_m1
Tgfb1	Thermofisher	Mm01178820_m1
Acta2	Thermofisher	Mm01546133_m1
Lox1	Thermofisher	Mm00454582_m1
Lox2	Thermofisher	Mm01325281_m1
Mmp2	Thermofisher	Mm00439498_m1
Mmp9	Thermofisher	Mm00442991_m1

Supplemental Table 2- Supplies and chemical assays used in this study

Category	Name	Supplier	Cat No.
Diets	Western HFD	Envigo	TD.88137
	Control LFD	Envigo	TD.06416
Chemical assays	AST assay kit	Thermofisher	TR70121
	ALT assay kit	Thermofisher	TR71121
	Free fatty acid kit	Sigma	MAK044-1KT
	Cholesterol assay kit	Thermofisher	TR13421
	Triglyceride assay kit	Thermofisher	TR22421
	Insulin ELISA kit	ALPCO	80-INSMR-CH10
	Glucose strip	Bayer HealthCare	7090G

Supplemental Table 3-Plasma peptides changed in post-LT fibrosis

Protein	Accession	Start	End	Sequence	Log2FC	-Log10PV
A1AG2	P19652	134	145	LDDEKNWGLSFY	-1.84	2.73
A2AP	P08697	42	53	EQVSPLTLLKLG	-3.34	8.75
ACTA	P62736	194	202	ILTERGYSF	-1.54	1.33
ACTB	P60709	2	15	D(+42.01)DDIAALVVD(+21.98)NGSG	4.66	2.40
		2	11	D(+42.01)DDIAALVVD	1.39	2.58
		20	36	GFAGDDAPRAVFPSIVG	1.15	2.76
		24	36	DDAPRAVFPSIVG	-3.96	5.12
		49	67	Q(-17.03)KDSYVGDEAQSKRGILTL	-1.87	3.00
		54	66	VGDEAQSKRGILT	2.59	2.48
		99	113	EEHPVLLTEAPLNPK	-2.04	1.88
		162	174	TVPIYEGYALPHA	-1.66	1.53
ANDR	P10275	447	480	YGPCGGGGGGGGGGGGGGGGGGGGGGEAGAVAP	1.63	1.37
		455	477	GGGGGGGGGGGGGGGGGGGEAGA	-4.66	9.73
AP2B1	P63010	678	694	APSPTPAVVSSGLNDLF	-1.96	3.55
APOC3	P02656	82	99	SEFWDLDPEVRPTSAVAA	4.94	5.45
APOF	Q13790	314	326	SYDLDPGAGSLEI	-3.36	3.70
BIN2	Q9UBW5	263	272	SPPVRTATVS	2.38	4.42
		536	565	QLQVSMVPENNNLTAPEPQEEVSTSENPQL	3.71	7.66
		541	565	MVPENNNLTAPEPQEEVSTSENPQL	-1.11	2.40
C1QB	P02746	83	101	PGNPGKVGPKGPMGPKGGP	6.19	11.83
CALD1	Q05682	119	130	KEFDPTITDASL	-1.48	2.24
		120	134	EFDPTITDASLSLPS	-3.78	9.02
		716	722	WEKGNVF	2.61	5.77
CASS4	Q9NQ75	164	180	ASLPTLPSQVYDVPTQH	3.88	7.11
CAVN2	O95810	21	31	EKPSSPSPM(+15.99)PS	3.36	2.45
		234	243	SSLKKVDSLK	1.32	1.42
		235	247	SLKKVDSLKKAFS	6.61	11.83
		288	294	SPFKVSP	2.42	1.82
CD99	P14209	175	184	EPAVQRTLLE	1.11	1.37
CFAB	P00751	239	257	TETIEGVDAEDGHGPGEQQ	2.86	7.30
CMGA	P10645	99	131	GFEDELSEVLENQSSQAELKEAVEEPSSKDVME	-4.73	10.05
CMIP	Q8IY22	720	732	ETPVTDAGLLALS	2.07	1.98
CNN2	Q99439	2	15	S(+42.01)S(+79.97)TQFNKGPSYGLS	1.92	2.23
CNST	Q6PJW8	320	334	TESSKESQHTVEPLG	3.14	2.67
CO1A1	P02452	371	397	GEPGPPGPAGAAGPAGNPGADGQPGAK	1.54	2.18
		546	558	SP(+15.99)GPDGKTGPP(+15.99)GP	2.29	6.16
		818	844	GADGQPGAKGEP(+15.99)GDAGAKGDAGPP(+15.99)GPA	5.70	10.98
		819	844	ADGQPGAKGEP(+15.99)GDAGAKGDAGPP(+15.99)GPA	2.39	3.92
		820	843	DGQP(+15.99)GAKGEPGDAGAKGDAGPPGP(+15.99)	7.13	11.17
		1151	1203	GKDGLNGLPGPIGPPGPRGRTGDAGPVGPPGPPGPPGPPGPPSAGFDFSFLPQ	-10.05	20.03
CO3	P01024	955	982	EGVQKEDIPPADLSDQVPDTESETRILL	2.31	2.04

Protein	Accession	Start	End	Sequence	Log2FC	-Log10PV
		969	987	DQVPDTESETRILLQGTPV	3.02	6.79
		1321	1343	SEETKENEGFTVTAEGKGQGTLS	7.10	6.49
		1321	1341	SEETKENEGFTVTAEGKGQGT	5.83	11.83
		1322	1336	EETKENEGFTVTAEG	4.56	3.06
CO3A1	P02461	500	510	P(+15.99)GEKGPAGERG	2.54	2.75
		1151	1166	DGTS(-18.01)GHPGPIGPP(+15.99)GPR(+28.03)	8.60	8.63
CO4A	P0C0L4	957	979	TLEIPGNSDPNM(+15.99)IPDGDFNSYVR	2.73	6.41
		1337	1349	NGFKSHALQLNNR	1.77	1.54
		1429	1449	DDPDAPLQPVTPLQLFEGRR(+0.98)N	3.43	5.28
		1429	1439	DDPDAPLQPVT(-18.01)	5.04	8.26
		1431	1443	PDAPLQPVTPLQL	1.05	2.30
CO4A2	P08572	365	386	PGFPGAQGEPGSQGEPGDPGLP	1.96	4.04
CO7A1	Q02388	1783	1797	LDGKPGAAGPSGPNG	-4.44	5.53
COMA1	Q8NFW1	911	922	APGAAGNPGAPG	1.31	1.57
CPNS1	P04632	39	60	GGGGGGGGGGGGGGGGGT(-18.01)AMR(+21.98)	-2.64	8.09
CRKL	P46109	214	239	TPLPAVSGSPGAAITPLPSTQNGPVF	1.03	2.30
DOK3	Q7L591	358	369	AEPGPQSLPLLL	2.79	2.81
DREB	Q16643	33	61	TYEDGSDDLKLAASGEGGLQELSGHFENQ	2.04	3.59
		467	490	NVPPAATSLIDLWPGNGEGASTLQ	1.92	1.54
		468	490	VPPAATSLIDLWPGN(+.98)GEGASTLQ	-1.16	1.65
		468	490	VPPAATSLIDLWPGNGEGASTLQ	1.11	1.62
		473	490	TSLIDLWPGNGEGASTLQ	-2.70	4.60
EPN4	Q14677	322	338	SSGDLVDLFDGTSQSTG	-1.58	4.10
ESYT2	A0FGR8	311	319	MPLVGALSI	-3.28	3.14
FBLN3	Q12805	112	131	VLPGGGFVASAAAVAGPEMQ	-1.52	1.51
FHOD1	Q9Y613	365	389	RRSLEGGGCPARAPEPGPTGPASPV	6.47	8.09
FIBA	P02671	20	35	ADS(+79.97)GEGD(-18.01)FLAEGGGVR	-3.47	9.97
		20	34	AD(-18.01)SGEGDFLAEGGGV	5.10	6.80
		24	34	EGDFLAEGGGV	-2.96	6.53
		26	35	DFLAEGGGVR	-1.43	2.19
		29	38	AEGGGVRGPR	3.65	6.43
		229	238	VPDLVPGNFK	2.22	3.84
		231	237	DLVPGNF	3.00	2.63
		260	269	ELERPGGNEI	-2.08	1.68
		272	296	GGSTSYGTGSETESPRNPSSAGSWN	5.61	7.76
		300	332	SGPGSTGNRNPGSSGTGGTATWKPGSSGPGSTG	-1.53	5.44
		303	328	GSTGN(+0.98)RNPGSSGTGGTATWKPGSSGP	-4.50	5.36
		330	361	S(+27.99)TGSWNSGSSGTGSTGNQNPGSPRPGSTGTWN	-2.70	3.25
		380	407	SGSTGQWHSESGSFRPDSPGSGNARPNN	6.45	10.18
		380	406	SGSTGQWHSESGSFRPDSPGSGNARPN.N	6.29	10.61
		392	424	SFRPDSPGSGNARPNNPDWGTFEEVSGNVSPGT	-1.18	2.59
		406	425	NNPDWGTFEEVSGNVSPGTR	-1.46	1.86
		426	443	REYHTEKLVTSKGDKELR	1.15	1.68

Protein	Accession	Start	End	Sequence	Log2FC	-Log10PV
		430	441	TEKLVTSKGD(-18.01)KE	-1.52	1.73
		521	536	DTASTGKTFPGFFSPM(+15.99)	1.34	1.67
		525	535	TGKTFPGFFSP	5.30	8.33
		537	557	LGEFVSETESRGSESGIFTNT	1.48	2.83
		538	557	GEFVSETESRGSESGIFTNT	-1.41	3.36
		542	600	SETESRGSESGIFTNTKESSSHHPGIAEFPSRGKSSSYSKQFTSSTSYNRGDSTFESKS	7.07	7.25
		542	598	SETESRGSESGIFTNTKESSSHHPGIAEFPSRGKSSSYSKQFTSSTSYNRGDSTFES	6.79	7.76
		542	587	SETESRGSESGIFTNTKESSSHHPGIAEFPSRGKSSSYSKQFTSST	7.87	4.15
		542	582	SETESRGSESGIFTNTKESSSHHPGIAEFPSRGKSSSYSKQ	7.09	6.42
		542	577	SETESRGSESGIFTNTKESSSHHPGIAEFPSRGKSS	7.61	4.89
		542	576	SETESRGSESGIFTNTKESSSHHPGIAEFPSRGKS	2.12	2.82
		542	574	SETESRGSESGIFTNTKESSSHHPGIAEFPSRG	5.63	2.87
		542	570	SETESRGSESGIFTNTKESSS(-18.01)HHPGIAEF	6.69	8.36
		542	566	SETESRGSESGIFTNTKESSSHHPG	3.87	2.77
		542	564	SETESRGSESGIFTNTKESSSHH	5.81	11.83
		542	562	SETESRGSESGIFTNTKESSS	2.82	8.33
		542	556	SETESRGSESGIFTN	6.05	11.56
		543	556	ETESRGSESGIFTN	-3.15	2.70
		543	555	ETESRGSESGIFT	4.64	4.89
		551	566	SGIFTNTKESSSHHPG(+15.99)	3.24	2.03
		552	568	GIFTNTKESSSHHP(+15.99)GIA	2.40	4.80
		559	578	ESSSHHPGIAEFPSRGKSSS	2.27	2.21
		576	596	SSSYSKQFTSSTSYNRGD(-18.01)STF	1.71	1.97
		582	604	Q(-17.03)FTSSTSYNRGDSTFESKSYKMA	4.85	9.13
		582	598	QFTSSTSYNRGDSTFES	3.16	6.27
		584	596	TSSTSYNRGDSTF	3.00	2.49
		586	603	STSYNRGDSTFESKSYKM(+15.99)	-2.81	5.61
		590	601	NR(+0.98)GDSTFESKSY	5.84	11.83
		597	619	ESKSYKM(+15.99)ADEAGSEADHEGTHST	5.27	7.94
		600	629	SYKMADEAGSEADHEGTHSTKRGHAKSRPV	4.96	3.75
		602	624	KMADEAGSEADHEGTHSTKRGHA	5.62	10.98
		602	622	KM(+15.99)ADEAGSEADHEGTHSTKRG	2.26	2.40
		603	629	MADEAGSEADHEGTHSTKRGHAKSRPV	-3.32	3.51
		605	629	DEAGSEADHEGTHSTKR(+0.98)GHAKSRPV	3.33	2.48
		605	628	DEAGSEADHEGTHSTKR(+0.98)GHAKSRP	1.50	2.28
		607	629	AGSEADHEGTHSTKR(+0.98)GHAKSRPV	2.60	2.05
		607	619	AGSEADHEGTHST	3.85	5.83
		609	624	SEADHEGTHSTKRGHA	2.68	4.49
		610	624	EADHEGTHSTKRGHA	2.32	1.90
FIBB	P02675	31	50	Q(-17.03)GVNDNEEGFFSAR(+0.98)GHRPLD	5.61	10.31
		31	50	QGVNDNEEGFFS(-18.01)AR(+0.98)GHRPLD	7.86	10.02
		31	44	Q(-17.03)GVN(+0.98)DNEEGFFSAR	1.89	2.52
		33	45	VNDNEEGFFSARG	1.65	2.23
		52	71	KREEAPSLRP(+15.99)APPPISGGGY	-1.28	2.49

Protein	Accession	Start	End	Sequence	Log2FC	-Log10PV
FIBG	P02679	437	453	EHPAETEYDSLYPEDDL	4.89	5.28
FLNA	P21333	1048	1060	DGVPVPGSPFPLE	2.01	2.05
FRM4B	Q9Y2L6	584	601	DTTTYDDPSDAFTFPGQR	1.97	3.02
FYB1	O15117	44	58	NASPPAGPSNVPKFG	2.05	2.60
FZD8	Q9H461	342	368	GGAPGAGGAGGAGGAAAGAGAAGAGAG	6.30	12.01
GELS	P06396	420	431	VPFDAATLHTST	1.85	2.63
		420	429	VPFDAATLHT	1.46	1.44
H12	P16403	2	21	S(+42.01)ETAPAAPAAAPPAEKAPVK	5.49	9.86
H14	P10412	2	21	S(+42.01)ETAPAAPAAPAPAEKTPVK	1.36	2.11
H15	P16401	2	20	S(+42.01)ETAPAETATPAPVEKSPA	1.48	1.68
HSPB1	P04792	34	42	GLPRLPEEW	1.56	1.37
		177	185	NEITIPVTF	2.58	3.74
		190	204	QLGGPEAAKSDETAA	1.39	1.51
IGF2	P01344	93	110	DVSTPPTVLPDNFPRYPV(-0.98)	3.67	4.04
INS	P01308	57	86	EAEDLQVGQVELGGGPGAGSLQPLALEGSL	-4.38	4.95
		57	79	EAEDLQVGQVELGGGPGAGSLQP	3.89	2.76
INSM1	Q01101	135	158	AALLGGGGGGGASGAGGGGTCGGD	3.71	3.31
IRF1	P10914	101	109	KGSSAVRVY	-1.06	2.21
ITIH4	Q14624	488	500	GSEMVVAGKLQDR	-1.46	1.33
		489	500	SEM(+15.99)VVAGKLQDR	4.08	7.30
		617	625	N(+0.98)VHSGSTFF	3.95	8.09
		635	644	PKPEASFSPR	-2.13	2.95
		647	659	WNRQAGAAGSRM(+15.99)N	5.83	4.49
		660	666	FRPGVLS	4.21	6.97
		663	683	GVLSSRQLGLP(+15.99)GPPDVPDHAA	1.81	5.96
		669	684	Q(-17.03)LGLPGPPDVPDHAAY	-1.00	1.56
		673	686	PGPPDVPDHAAYHP	1.52	1.68
		673	682	PGPPDVPDHA	-2.58	4.06
JUND	P17535	153	173	LGAGAAAAAAAAAAGGPSGTA	1.21	1.68
K0513	O60268	238	249	TENVKGFFGGLE	-1.31	1.51
KLD10	Q6PID8	17	36	AAGAGGGGSGAGGGSGGSGG	-1.52	3.51
LASP1	Q14847	160	172	Q(-17.03)PHHIPTSAPVYQ	1.63	2.13
LEGL	Q3ZCW2	2	22	A(+42.01)GSVADSDAVVKLDDGHLN(+0.98)NS	1.49	2.71
		7	23	DSDAVVKLDDGHLNNSL	-1.07	1.96
LPP	Q93052	182	212	PQPAPQAGPIPVAPIGTLKPQPQPVPASYTT	2.34	3.56
LRBA	P50851	1568	1583	SENENVSLSEITPAAF	-1.53	1.95
LTBP1	Q14766	821	834	GQPQLSPGISTIHL	-1.81	2.02
MAGD4	Q96JG8	83	90	QTLVEALQ	1.41	2.67
MGP	P08493	21	36	ESHESMESYELNPFIN	2.16	1.70
MMRN1	Q13201	296	311	AESHTAVGRGVAEQQQ	2.21	3.22
MOES	P26038	469	495	TPHVAEPAENEQDEQDENGAEASADLR	2.63	4.05
		533	545	RDESKKTANDM(+15.99)IH	3.21	4.25

Protein	Accession	Start	End	Sequence	Log2FC	-Log10PV
MYH9	P35579	2	11	A(+42.01)QQAADKYLY	7.50	10.24
		1936	1955	RKGAGDGSDEEVDGKADGAE	-1.14	1.53
NDUC2	O95298	107	115	TYGEIFEKF	3.43	5.61
OIT3	Q8WWZ8	520	545	GAGGEDSAGLQGQTLTGGPIRIDWED	-4.73	8.83
PDLI1	O00151	137	173	ARVITNQYNNPAGLYSSENISNFNNALESKTAASGVE	1.68	2.76
		152	173	SSENISNFNN(+0.98)ALESKTAASGVE	2.93	7.30
		162	173	ALESKTAASGVE	-3.74	7.39
		170	181	SGVEANSRPLDH	1.36	1.87
		225	238	SEEKGDPNKPSGFR	2.33	2.64
PDLI5	Q96HC4	161	174	ASPSPVAAVTPPLF	2.66	1.6
		164	174	SPVAAVTPPLF	2.33	1.68
PDLI7	Q9NR12	100	113	DPPRYTFAPSVSLN	4.60	7.42
PIGR	P01833	604	639	LFAEEKAVADTRDQADGSRASVDSGSSEEQGGSSRA	1.68	1.81
PLEC	Q15149	3116	3125	SLVPAAELLE	-3.06	5.61
PLEK	P08567	283	292	AEDPLGAIHL	-7.37	16.16
PRRC1	Q96M27	164	180	TGLLPTPITQQASLTSL	2.67	5.61
PTN12	Q05209	568	591	LTPSPTTQVETPDLVDHDNTSPLF	-1.09	1.42
RTN4	Q9NQC3	97	124	APPVAPERQPSWDPSPVSSTVPAPSPLS(+21.98)	3.08	3.20
S10A8	P05109	18	26	KYSLIKGNF	2.69	2.87
SAA2	P0DJI9	109	122	DPNHFRPAGLPEKY	-3.43	11.83
SCAM2	O15127	31	53	QGGLAEFNPFSETNAATTVPVTQ	-1.00	2.25
SKAP2	O75563	253	277	SQPIDDEIYEELPEEEEDSAPVKVE	-1.16	2.66
SKOR1	P84550	292	319	GGSGGQGKGGAGGGGGGGPGCGAEM(+15.99)APG	3.55	3.40
SRC8	Q14247	130	141	DRVDQSAVGFEY	-1.82	4.11
SYUA	P37840	32	47	KTKEGVLYVGSKTKEG	1.46	4.78
TAGL2	P37802	2	12	A(+42.01)NRGPAYGLSR	3.98	4.89
		2	11	A(+42.01)NRGPAYGLS(-18.01)	2.82	4.60
		180	199	TNRGASQAGM(+15.99)TGYGM(+15.99)PRQIL	-3.09	3.20
		180	192	TNRGASQAGM(+15.99)TGY	4.13	3.50
		181	197	NRGASQAGMTGYGMPRQ	4.39	7.76
TBA1B	P68363	29	57	GIQPDGQMPSDKTIGGGDDSFNTFFSETG	-1.58	3.00
		37	57	PSDKTIGGGDDSFNTFFSETG	-3.23	3.59
		66	79	VFVDLEPTVIDEVR	1.82	2.16
		169	178	FSIYPAPQVS	1.83	2.86
		230	240	LISQIVSSITA	1.71	5.16
		276	285	ISAEKAYHEQ	1.10	1.31
		283	293	HEQLSVAEITN	-1.16	1.77
		381	392	TTAIAEAWARLD	3.15	5.88
		382	392	TAIAEAWARLD	-2.68	9.73
		430	450	KDYEEVGVDS(-18.01)VEGEGEEEGEE	4.68	9.73
TBA1C	Q9BQE3	379	392	SNTTAVAEAWARLD	-1.64	2
		430	449	KDYEEVGADSADGEDEGEEY	1.26	2.24
TBA4A	P68366	38	52	SDKTIGGGDDSFTTF	-1.65	1.66

Protein	Accession	Start	End	Sequence	Log2FC	-Log10PV
		430	446	KDYEEVGIDSYEDEDEG	1.37	2.22
		437	448	IDSYEDEDEGEE	1.00	1.51
TBA8	Q9NY65	41	57	SKINDDDSFTTFFSETG	-1.18	2.43
		43	57	INDDDSFTTFFSETG	2.97	5.21
		43	52	INDDDSFTTF	-1.28	2.67
		276	285	ISAEKAYHEQ	1.10	1.31
TBB1	Q9H4B7	107	118	TEGAELIENVLE	1.90	1.89
		168	192	SVMPSPKVSDTVVEPYNAVLSIHQL	-1.26	1.45
		168	187	SVMPSPKVSDTVVEPYNAVL	-1.48	1.55
		218	232	TTPTYGDLNHLVSLT	2.82	2.4
		403	419	MDINEFGEAENNIHDLV	1.00	2.24
TBB5	P07437	14	25	N(+0.98)QIGAKFWEVIS	-1.30	2.64
		16	25	IGAKFWEVIS	2.47	3.29
		106	121	YTEGAELVDSVLDVVR	3.44	5.59
		123	136	EAES(-18.01)CD(+15.99)CLQGFQLT	2.03	2.43
		224	238	DLNHLVSATMSGVTT	-1.40	2.90
		420	443	SEYQQYQDATAEEEED(-18.01)FGEEAEEE	3.61	6.83
		420	441	SEYQQYQDATAEEEED(-18.01)FGEEAE	3.40	7.66
		423	444	Q(-17.03)QYQDATAEEEEDFGEEAEEEA	1.18	1.40
TCRG1	O14776	255	271	AVGASTPTTSSPAPAVS	2.41	2.53
TLN1	Q9Y490	409	427	FGLEGDEESTMLEDSVSPK	-1.48	2.76
		436	446	YNRVGKVEHGS(-18.01)	-2.30	3.61
		873	891	AAKGAAAHPDSEEQQQRLR	1.08	1.92
		1531	1541	EVANSTANLVK	-1.24	2.11
		1560	1575	AATAPLLEAVDNLSAF	1.55	1.86
		1569	1581	VDNLSAFASNPEF	4.64	7.87
		2512	2519	ERELEEAR	3.90	1.63
TMOD3	Q9NYL9	170	177	GEKILPVF	-1.73	3.01
TRML1	Q86YW5	218	235	DSGPAAELPLDVPHIRLD	4.89	7.51
		234	251	LDSPPSFDNTTYTSLPLD	2.18	5.53
		246	274	TSLPLDSPSGKPSLPAPSSLPPLPPKVLV	3.83	6.42
		247	273	SLPLDSPSGKPSLPAPSSLPPLPPKVL	5.63	11.83
TYB10	P63313	2	18	ADK(+42.02)PDMGEIASFDKAKL	-4.65	5.57
TYB4	P62328	2	27	SDK(+42.01)PDMAEIEKFDKSKLKKT(-18.01)ETQEKN	7.13	11.01
		2	25	S(+42.01)DKPDM(+15.99)AEIEKFDKSKLKKTETQE	7.66	10.06
		2	23	S(+42.01)DKPDMAEIEKFDKSKLKKTET	2.72	2.43
		2	22	SDK(+42.02)PDMAEIEKFDKSKLKKTE	3.79	2.44
		2	22	S(+42.01)DKPDMAEIEKFDKSKLKKTE	8.83	8.36
		26	38	KNPLPSKET(-18.01)IEQE	2.83	5.22
UBQL1	Q9UMX0	293	308	GNPFASLVSNTSSGEG	3.65	7.25
URP2	Q86UX7	338	352	APTDVLDSLTTIPEL	-1.21	1.54
		341	352	DVLDSLTTIPEL	-2.95	6.95
VASP	P50552	128	141	SVPNGPSPEEVEQQ	2.55	2.81

Protein	Accession	Start	End	Sequence	Log2FC	-Log10PV
VIME	P08670	30	38	YVTTSTRTY	-1.86	1.44
VTNC	P04004	94	115	GGPSLTSDLQAQSKGNPEQTPV	2.80	4.85
		97	115	SLTSDLQAQSKGNPEQTPV	6.45	9.82
		108	128	GNPEQTPVLKPEEEAPAPEVG	1.84	2.69
		117	135	KPEEEAPAPEVGASKPEGI	4.84	8.94
		119	135	EEEAPAPEVGASKPEGI	6.99	7.23
WDR44	Q5JSH3	63	72	IIESIIEESQ	2.21	2.54
ZYX	Q15942	247	279	LANTQPRGPPASSPAPAPKFSPVTPKFTPVASK	1.01	1.49
		345	360	PGAPGPLTLKEVEELE	-3.39	8.33

Log2FC and -Log10PV are in comparison to values from patients that did not progress to >F2 fibrosis. Start and end of the peptide are based on the FASTA protein sequence associated with the accession number. Mass addition (e.g., +42.02) and mass loss (e.g., -18.01) denotes chemical modification of the amino acid, corresponding to the following:

-18.01	dehydration
-17.03	pyroglutamic acid
-0.98	amidation
+0.98	deamidation
+15.99	oxidation
+18.01	hydration
+21.98	sodiation
+27.99	formylation
+28.03	dimethylation
+42.01	acetylation
+42.02	guanidiation
+43.01	carbamylation
+79.97	phosphorylation
+156.12	4-hydroxynonenal
+162.05	hexose

Supplemental Table 4-GO terms for Cellular Component (GO:0005575) of peptides changed in post-LT fibrosis

Term ID #	Term Description	Count	Enrichment	-Log10PV
**Increased in post LT fibrosis**				
GO:0015629	actin cytoskeleton	20	0.99	11.06
GO:0034774	secretory granule lumen	18	1.07	11.06
GO:0005856	cytoskeleton	37	0.58	10.75
GO:0099503	secretory vesicle	26	0.76	10.75
GO:0030141	secretory granule	24	0.79	10.36
GO:0005938	cell cortex	14	1.11	9.42
GO:0005747	mitochondrial respiratory chain complex I	8	1.56	8.21
GO:0043229	intracellular organelle	85	0.17	8.21
GO:0030863	cortical cytoskeleton	9	1.39	8.15
GO:0031410	cytoplasmic vesicle	34	0.51	8.15
GO:0005737	cytoplasm	81	0.18	7.88
GO:0099568	cytoplasmic region	15	0.89	7.72
GO:0005576	extracellular region	34	0.46	7.01
GO:0005622	intracellular	89	0.12	6.7
GO:0005788	endoplasmic reticulum lumen	12	0.93	6.45
GO:0043232	intracellular non-membrane-bounded organelle	43	0.35	6.45
GO:0099081	supramolecular polymer	19	0.66	6.38
GO:0098644	complex of collagen trimers	5	1.74	6.03
GO:0032991	protein-containing complex	46	0.31	5.7
GO:0012505	endomembrane system	43	0.32	5.55
GO:0005581	collagen trimer	7	1.22	5.49
GO:0030864	cortical actin cytoskeleton	6	1.38	5.48
GO:0031091	platelet alpha granule	7	1.21	5.42
GO:0002102	podosome	5	1.54	5.26
GO:0099512	supramolecular fiber	17	0.61	5.14
GO:0031093	platelet alpha granule lumen	6	1.27	4.94
GO:0005925	focal adhesion	7	1.04	4.4
GO:0005924	cell-substrate adherens junction	7	1.03	4.36
GO:0030054	cell junction	17	0.55	4.36
GO:0001726	ruffle	7	0.95	3.89
GO:0005577	fibrinogen complex	3	1.9	3.89
GO:0042641	actomyosin	5	1.22	3.89
GO:0005615	extracellular space	17	0.5	3.74
GO:0005911	cell-cell junction	10	0.72	3.74
GO:0005912	adherens junction	8	0.82	3.66
GO:0005583	fibrillar collagen trimer	3	1.76	3.62
GO:0005623	cell	90	0.07	3.52
GO:0062023	collagen-containing extracellular matrix	6	0.94	3.33
GO:0070013	intracellular organelle lumen	42	0.23	3.33
GO:0043227	membrane-bounded organelle	71	0.12	3.17
GO:0001725	stress fiber	4	1.23	3.16
GO:0005584	collagen type I trimer	2	2.32	3.14
GO:0071944	cell periphery	41	0.22	2.85
GO:0101002	ficolin-1-rich granule	6	0.83	2.8
GO:0030478	actin cap	2	2.02	2.77
GO:0005743	mitochondrial inner membrane	9	0.62	2.74
GO:0005886	plasma membrane	40	0.21	2.72
GO:0031966	mitochondrial membrane	11	0.53	2.7
GO:0031252	cell leading edge	8	0.66	2.68
GO:0031012	extracellular matrix	7	0.72	2.66
GO:0005874	microtubule	8	0.64	2.59
GO:0005587	collagen type IV trimer	2	1.85	2.55
GO:0034363	intermediate-density lipoprotein particle	2	1.85	2.55
GO:0042995	cell projection	20	0.33	2.43

Term ID #	Term Description	Count	Enrichment	-Log10PV
GO:0042582	azurophil granule	5	0.83	2.39
GO:0034366	spherical high-density lipoprotein particle	2	1.67	2.32
GO:0098794	postsynapse	8	0.59	2.32
GO:0120025	plasma membrane bounded cell projection	19	0.32	2.24
GO:0001931	uropod	2	1.51	2.06
GO:0042627	chylomicron	2	1.51	2.06
GO:0045202	synapse	11	0.44	2.03
GO:0031967	organelle envelope	13	0.38	1.93
GO:0014069	postsynaptic density	5	0.71	1.93
GO:1904813	ficolin-1-rich granule lumen	4	0.83	1.92
GO:0031143	pseudopodium	2	1.39	1.9
GO:0005905	clathrin-coated pit	3	1	1.88
GO:0099513	polymeric cytoskeletal fiber	9	0.47	1.88
GO:0030016	myofibril	5	0.69	1.87
GO:0030425	dendrite	8	0.5	1.87
GO:0005783	endoplasmic reticulum	17	0.3	1.82
GO:0016020	membrane	53	0.12	1.81
GO:0034361	very-low-density lipoprotein particle	2	1.32	1.81
GO:0030659	cytoplasmic vesicle membrane	9	0.42	1.61
GO:0036477	somatodendritic compartment	9	0.41	1.59
GO:0070820	tertiary granule	4	0.71	1.59
GO:0031970	organelle envelope lumen	3	0.86	1.57
GO:0005604	basement membrane	3	0.84	1.52
GO:0035578	azurophil granule lumen	3	0.84	1.51
GO:0098796	membrane protein complex	11	0.34	1.49
GO:0030027	lamellipodium	4	0.66	1.44
GO:0005720	nuclear heterochromatin	2	1.08	1.42
GO:0032993	protein-DNA complex	4	0.65	1.42
GO:0000786	nucleosome	3	0.77	1.37
GO:0048471	perinuclear region of cytoplasm	8	0.4	1.37
GO:0098805	whole membrane	14	0.28	1.37
GO:0005773	vacuole	8	0.39	1.34
GO:0015630	microtubule cytoskeleton	11	0.32	1.33
**Decreased in post LT fibrosis**				
GO:0005856	cytoskeleton	22	0.63	6.58
GO:0099080	supramolecular complex	15	0.83	6.48
GO:0015629	actin cytoskeleton	11	1.01	6.1
GO:0099512	supramolecular fiber	14	0.81	5.88
GO:0031093	platelet alpha granule lumen	5	1.47	4.35
GO:0002102	podosome	4	1.73	4.26
GO:0005938	cell cortex	7	1.08	4.26
GO:0034774	secretory granule lumen	8	1	4.26
GO:0005577	fibrinogen complex	3	2.18	4.26
GO:0005576	extracellular region	19	0.48	4.09
GO:0005737	cytoplasm	43	0.18	4.09
GO:0043232	intracellular non-membrane-bounded organelle	24	0.38	3.89
GO:0030141	secretory granule	10	0.68	3.31
GO:0030863	cortical cytoskeleton	4	1.32	3.17
GO:0099568	cytoplasmic region	7	0.84	3.08
GO:0005622	intracellular	46	0.11	2.59
GO:0031410	cytoplasmic vesicle	15	0.43	2.55
GO:0043292	contractile fiber	5	0.94	2.54
GO:0031252	cell leading edge	6	0.81	2.48
GO:0005874	microtubule	6	0.79	2.43
GO:0005924	cell-substrate adherens junction	4	1.07	2.43
GO:0005925	focal adhesion	4	1.08	2.43
GO:0042641	actomyosin	3	1.28	2.32
GO:0001726	ruffle	4	0.99	2.2

Term ID #	Term Description	Count	Enrichment	-Log10PV
GO:0043229	intracellular organelle	41	0.13	2.12
GO:0099513	polymeric cytoskeletal fiber	7	0.64	2.09
GO:0098644	complex of collagen trimers	2	1.62	2.06
GO:0005615	extracellular space	9	0.5	1.9
GO:0030016	myofibril	4	0.87	1.84
GO:0034364	high-density lipoprotein particle	2	1.46	1.79
GO:0005912	adherens junction	4	0.8	1.64
GO:0032991	protein-containing complex	21	0.24	1.64
GO:0031012	extracellular matrix	4	0.75	1.51
GO:0062023	collagen-containing extracellular matrix	3	0.92	1.5
GO:0015630	microtubule cytoskeleton	8	0.46	1.48
GO:0005788	endoplasmic reticulum lumen	4	0.73	1.46
GO:0001725	stress fiber	2	1.2	1.43
GO:0030864	cortical actin cytoskeleton	2	1.18	1.4

Supplemental Table 5-GO terms for Biological Process (GO:0008150) of peptides changed in post-LT fibrosis

Term ID #	Term Description	Count	Enrichment	-Log10PV
**Increased in post LT fibrosis**				
GO:0045055	regulated exocytosis	25	0.88	11.30
GO:0001775	cell activation	27	0.74	9.96
GO:0002576	platelet degranulation	13	1.33	9.96
GO:0016192	vesicle-mediated transport	34	0.62	9.96
GO:0016043	cellular component organization	56	0.36	8.53
GO:0022607	cellular component assembly	37	0.52	8.47
GO:0007010	cytoskeleton organization	23	0.71	7.62
GO:0006120	mitochondrial electron transport, NADH to ubiquinone	8	1.58	7.62
GO:0065003	protein-containing complex assembly	28	0.59	7.32
GO:0042775	mitochondrial ATP synthesis coupled electron transport	9	1.39	7.30
GO:0043933	protein-containing complex subunit organization	30	0.55	7.23
GO:0006996	organelle organization	40	0.43	7.01
GO:0030036	actin cytoskeleton organization	15	0.88	6.66
GO:0032981	mitochondrial respiratory chain complex I assembly	8	1.41	6.64
GO:0030168	platelet activation	9	1.2	5.97
GO:0034622	cellular protein-containing complex assembly	19	0.68	5.85
GO:1900026	positive regulation of substrate adhesion-dependent cell spreading	6	1.6	5.67
GO:0050776	regulation of immune response	19	0.66	5.58
GO:0097435	supramolecular fiber organization	13	0.85	5.43
GO:0048584	positive regulation of response to stimulus	29	0.47	5.39
GO:0051130	positive regulation of cellular component organization	21	0.59	5.27
GO:0002376	immune system process	31	0.44	5.21
GO:0002682	regulation of immune system process	23	0.54	5.07
GO:0051234	establishment of localization	43	0.33	5.00
GO:0007160	cell-matrix adhesion	8	1.15	4.98
GO:0006897	endocytosis	14	0.76	4.97
GO:0006810	transport	42	0.33	4.92
GO:0045321	leukocyte activation	18	0.63	4.87
GO:0051258	protein polymerization	7	1.25	4.87
GO:0042060	wound healing	13	0.77	4.70
GO:0009611	response to wounding	14	0.73	4.68
GO:0010770	positive regulation of cell morphogenesis involved in differentiation	8	1.09	4.65
GO:0051128	regulation of cellular component organization	29	0.42	4.51
GO:0043312	neutrophil degranulation	13	0.75	4.50
GO:0007015	actin filament organization	9	0.98	4.49
GO:0002274	myeloid leukocyte activation	14	0.71	4.49
GO:0010769	regulation of cell morphogenesis involved in differentiation	10	0.9	4.49
GO:0043062	extracellular structure organization	11	0.83	4.43
GO:0051179	localization	47	0.28	4.38
GO:0010811	positive regulation of cell-substrate adhesion	7	1.13	4.28
GO:0098657	import into cell	14	0.68	4.28
GO:0007596	blood coagulation	10	0.86	4.26
GO:0009205	purine ribonucleoside triphosphate metabolic process	9	0.93	4.26
GO:0022604	regulation of cell morphogenesis	12	0.76	4.25
GO:0010647	positive regulation of cell communication	23	0.47	4.19
GO:0009167	purine ribonucleoside monophosphate metabolic process	9	0.92	4.18
GO:0002443	leukocyte mediated immunity	14	0.67	4.18
GO:0023056	positive regulation of signaling	23	0.47	4.18
GO:0072376	protein activation cascade	6	1.23	4.16

Term ID #	Term Description	Count	Enrichment	-Log10PV
GO:2001233	regulation of apoptotic signaling pathway	11	0.78	4.04
GO:0022603	regulation of anatomical structure morphogenesis	17	0.57	4.04
GO:0006955	immune response	22	0.47	4.00
GO:0010810	regulation of cell-substrate adhesion	8	0.95	3.89
GO:0031347	regulation of defense response	14	0.64	3.89
GO:0050793	regulation of developmental process	28	0.39	3.85
GO:0034114	regulation of heterotypic cell-cell adhesion	4	1.6	3.80
GO:0050727	regulation of inflammatory response	10	0.79	3.80
GO:0009967	positive regulation of signal transduction	21	0.47	3.77
GO:0002252	immune effector process	16	0.56	3.66
GO:0048583	regulation of response to stimulus	37	0.3	3.66
GO:0006898	receptor-mediated endocytosis	8	0.91	3.64
GO:2000257	regulation of protein activation cascade	5	1.29	3.64
GO:0051235	maintenance of location	7	0.99	3.60
GO:0032101	regulation of response to external stimulus	16	0.55	3.52
GO:0010638	positive regulation of organelle organization	12	0.66	3.46
GO:0030198	extracellular matrix organization	9	0.81	3.46
GO:0060627	regulation of vesicle-mediated transport	11	0.68	3.30
GO:0050878	regulation of body fluid levels	11	0.68	3.28
GO:0006911	phagocytosis, engulfment	4	1.43	3.28
GO:0038063	collagen-activated tyrosine kinase receptor signaling pathway	3	1.85	3.28
GO:0051050	positive regulation of transport	15	0.55	3.28
GO:0070613	regulation of protein processing	6	1.04	3.24
GO:0051246	regulation of protein metabolic process	28	0.34	3.14
GO:0032879	regulation of localization	27	0.35	3.12
GO:0031639	plasminogen activation	3	1.76	3.09
GO:0007005	mitochondrion organization	10	0.7	3.06
GO:0032970	regulation of actin filament-based process	9	0.75	3.06
GO:0080134	regulation of response to stress	18	0.46	3.06
GO:0007155	cell adhesion	14	0.54	3.00
GO:1903035	negative regulation of response to wounding	5	1.12	3.00
GO:0034116	positive regulation of heterotypic cell-cell adhesion	3	1.69	2.96
GO:0034329	cell junction assembly	6	0.97	2.96
GO:0051247	positive regulation of protein metabolic process	20	0.42	2.96
GO:1902533	positive regulation of intracellular signal transduction	15	0.52	2.96
GO:0007229	integrin-mediated signaling pathway	5	1.1	2.89
GO:0008154	actin polymerization or depolymerization	4	1.29	2.85
GO:0032270	positive regulation of cellular protein metabolic process	19	0.43	2.85
GO:0071560	cellular response to transforming growth factor beta stimulus	6	0.96	2.85
GO:0001932	regulation of protein phosphorylation	18	0.44	2.82
GO:0090066	regulation of anatomical structure size	10	0.66	2.80
GO:0019220	regulation of phosphate metabolic process	20	0.4	2.77
GO:0022008	neurogenesis	19	0.42	2.77
GO:0002673	regulation of acute inflammatory response	5	1.06	2.74
GO:0032880	regulation of protein localization	14	0.51	2.74
GO:2001234	negative regulation of apoptotic signaling pathway	7	0.83	2.74
GO:0030195	negative regulation of blood coagulation	4	1.24	2.72
GO:0045597	positive regulation of cell differentiation	14	0.51	2.72
GO:0033043	regulation of organelle organization	16	0.46	2.70
GO:0034097	response to cytokine	15	0.48	2.70
GO:0045185	maintenance of protein location	5	1.04	2.70
GO:0051094	positive regulation of developmental process	17	0.44	2.70
GO:0090277	positive regulation of peptide hormone secretion	5	1.04	2.70
GO:0002253	activation of immune response	9	0.68	2.68
GO:0045595	regulation of cell differentiation	20	0.39	2.68

Term ID #	Term Description	Count	Enrichment	-Log10PV
GO:0048518	positive regulation of biological process	43	0.22	2.66
GO:0051621	regulation of norepinephrine uptake	2	2.32	2.66
GO:1903923	positive regulation of protein processing in phagocytic vesicle	2	2.32	2.66
GO:0009653	anatomical structure morphogenesis	22	0.37	2.64
GO:0030449	regulation of complement activation	4	1.21	2.64
GO:0065008	regulation of biological quality	32	0.28	2.62
GO:0110053	regulation of actin filament organization	7	0.8	2.62
GO:1902903	regulation of supramolecular fiber organization	8	0.73	2.62
GO:2001237	negative regulation of extrinsic apoptotic signaling pathway	5	1	2.59
GO:0043410	positive regulation of MAPK cascade	10	0.61	2.55
GO:0051239	regulation of multicellular organismal process	27	0.31	2.55
GO:0031399	regulation of protein modification process	20	0.38	2.54
GO:0032268	regulation of cellular protein metabolic process	25	0.33	2.54
GO:0042730	fibrinolysis	3	1.48	2.54
GO:0031532	actin cytoskeleton reorganization	4	1.17	2.52
GO:0060284	regulation of cell development	13	0.51	2.52
GO:0009150	purine ribonucleotide metabolic process	9	0.65	2.49
GO:0010903	negative regulation of very-low-density lipoprotein particle remodeling	2	2.15	2.49
GO:0043254	regulation of protein complex assembly	9	0.65	2.49
GO:0030162	regulation of proteolysis	12	0.53	2.48
GO:0048468	cell development	18	0.4	2.48
GO:0010646	regulation of cell communication	30	0.28	2.44
GO:0030334	regulation of cell migration	12	0.53	2.44
GO:0044087	regulation of cellular component biogenesis	13	0.5	2.44
GO:1902905	positive regulation of supramolecular fiber organization	6	0.85	2.44
GO:0048522	positive regulation of cellular process	39	0.22	2.41
GO:0002793	positive regulation of peptide secretion	7	0.75	2.40
GO:0040012	regulation of locomotion	13	0.49	2.40
GO:0007165	signal transduction	38	0.23	2.39
GO:0009966	regulation of signal transduction	28	0.29	2.39
GO:0010954	positive regulation of protein processing	3	1.4	2.39
GO:0023051	regulation of signaling	30	0.27	2.39
GO:0032507	maintenance of protein location in cell	4	1.12	2.39
GO:0048869	cellular developmental process	31	0.27	2.39
GO:0070527	platelet aggregation	3	1.4	2.39
GO:0002791	regulation of peptide secretion	9	0.62	2.36
GO:0030335	positive regulation of cell migration	9	0.62	2.36
GO:0040011	locomotion	15	0.44	2.36
GO:0050896	response to stimulus	54	0.16	2.36
GO:0031401	positive regulation of protein modification process	15	0.44	2.35
GO:0072378	blood coagulation, fibrin clot formation	3	1.37	2.32
GO:0045087	innate immune response	11	0.53	2.30
GO:0009605	response to external stimulus	22	0.33	2.29
GO:0035025	positive regulation of Rho protein signal transduction	3	1.35	2.29
GO:0048870	cell motility	13	0.48	2.29
GO:2000352	negative regulation of endothelial cell apoptotic process	3	1.35	2.29
GO:0045807	positive regulation of endocytosis	5	0.91	2.28
GO:0062013	positive regulation of small molecule metabolic process	5	0.91	2.28
GO:0045785	positive regulation of cell adhesion	8	0.65	2.26
GO:0045937	positive regulation of phosphate metabolic process	14	0.45	2.26
GO:0051495	positive regulation of cytoskeleton organization	6	0.79	2.26
GO:0016477	cell migration	12	0.49	2.24

Term ID #	Term Description	Count	Enrichment	-Log10PV
GO:0051493	regulation of cytoskeleton organization	9	0.6	2.24
GO:1902175	regulation of oxidative stress-induced intrinsic apoptotic signaling pathway	3	1.32	2.24
GO:0007167	enzyme linked receptor protein signaling pathway	11	0.52	2.24
GO:0010033	response to organic substance	26	0.29	2.23
GO:0001934	positive regulation of protein phosphorylation	13	0.46	2.22
GO:0007166	cell surface receptor signaling pathway	22	0.32	2.21
GO:0070887	cellular response to chemical stimulus	25	0.29	2.19
GO:1902531	regulation of intracellular signal transduction	19	0.36	2.19
GO:0042989	sequestering of actin monomers	2	1.85	2.18
GO:0043152	induction of bacterial agglutination	2	1.85	2.18
GO:0045921	positive regulation of exocytosis	4	1.03	2.18
GO:0051047	positive regulation of secretion	8	0.63	2.18
GO:0071310	cellular response to organic substance	22	0.32	2.18
GO:0071800	podosome assembly	2	1.85	2.18
GO:0098532	histone H3-K27 trimethylation	2	1.85	2.18
GO:1904035	regulation of epithelial cell apoptotic process	4	1.03	2.18
GO:0006928	movement of cell or subcellular component	16	0.4	2.17
GO:0007017	microtubule-based process	10	0.54	2.17
GO:0032989	cellular component morphogenesis	11	0.51	2.17
GO:0045907	positive regulation of vasoconstriction	3	1.28	2.17
GO:0072358	cardiovascular system development	9	0.58	2.17
GO:0032103	positive regulation of response to external stimulus	9	0.58	2.16
GO:0090207	regulation of triglyceride metabolic process	3	1.27	2.14
GO:0061024	membrane organization	11	0.5	2.13
GO:0071495	cellular response to endogenous stimulus	14	0.43	2.12
GO:0032102	negative regulation of response to external stimulus	7	0.68	2.11
GO:0032489	regulation of Cdc42 protein signal transduction	2	1.78	2.11
GO:0030155	regulation of cell adhesion	10	0.53	2.10
GO:0031325	positive regulation of cellular metabolic process	27	0.27	2.10
GO:1902042	negative regulation of extrinsic apoptotic signaling pathway via death domain receptors	3	1.24	2.10
GO:0044092	negative regulation of molecular function	14	0.42	2.08
GO:0019217	regulation of fatty acid metabolic process	4	0.99	2.08
GO:0030100	regulation of endocytosis	6	0.74	2.08
GO:0048523	negative regulation of cellular process	35	0.22	2.07
GO:0002224	toll-like receptor signaling pathway	4	0.99	2.06
GO:0032501	multicellular organismal process	46	0.17	2.06
GO:1903076	regulation of protein localization to plasma membrane	4	0.99	2.06
GO:0034371	chylomicron remodeling	2	1.72	2.04
GO:0050708	regulation of protein secretion	8	0.6	2.04
GO:0071394	cellular response to testosterone stimulus	2	1.72	2.04
GO:0006950	response to stress	28	0.26	2.04
GO:0051240	positive regulation of multicellular organismal process	17	0.36	2.04
GO:0030199	collagen fibril organization	3	1.21	2.03
GO:0007154	cell communication	39	0.2	2.02
GO:0009893	positive regulation of metabolic process	28	0.25	2.02
GO:0051173	positive regulation of nitrogen compound metabolic process	26	0.27	2.02
GO:0048699	generation of neurons	16	0.37	2.01
GO:2001242	regulation of intrinsic apoptotic signaling pathway	5	0.82	2.01
GO:0030154	cell differentiation	29	0.25	2.00
GO:0050714	positive regulation of protein secretion	6	0.72	2.00
GO:0042221	response to chemical	33	0.22	2.00
GO:0090087	regulation of peptide transport	10	0.51	1.99
GO:0016584	nucleosome positioning	2	1.67	1.98
GO:0043589	skin morphogenesis	2	1.67	1.98

Term ID #	Term Description	Count	Enrichment	-Log10PV
GO:1905475	regulation of protein localization to membrane	5	0.81	1.97
GO:0051346	negative regulation of hydrolase activity	8	0.58	1.97
GO:0051641	cellular localization	21	0.31	1.97
GO:0010038	response to metal ion	7	0.64	1.96
GO:0051049	regulation of transport	18	0.34	1.96
GO:0120036	plasma membrane bounded cell projection organization	13	0.42	1.96
GO:0031032	actomyosin structure organization	4	0.94	1.94
GO:0031647	regulation of protein stability	6	0.7	1.93
GO:0006959	humoral immune response	6	0.7	1.92
GO:0017157	regulation of exocytosis	5	0.79	1.92
GO:0034378	chylomicron assembly	2	1.62	1.92
GO:0070486	leukocyte aggregation	2	1.62	1.92
GO:0007399	nervous system development	21	0.3	1.92
GO:0045862	positive regulation of proteolysis	7	0.63	1.92
GO:0048585	negative regulation of response to stimulus	16	0.36	1.86
GO:0001568	blood vessel development	8	0.56	1.84
GO:0050790	regulation of catalytic activity	21	0.29	1.83
GO:0002764	immune response-regulating signaling pathway	7	0.61	1.82
GO:0006956	complement activation	3	1.11	1.82
GO:0010896	regulation of triglyceride catabolic process	2	1.54	1.82
GO:0033700	phospholipid efflux	2	1.54	1.82
GO:0043149	stress fiber assembly	2	1.54	1.82
GO:0051222	positive regulation of protein transport	7	0.61	1.82
GO:1903532	positive regulation of secretion by cell	7	0.61	1.82
GO:0010604	positive regulation of macromolecule metabolic process	26	0.25	1.80
GO:0006909	phagocytosis	5	0.75	1.79
GO:0071363	cellular response to growth factor stimulus	8	0.55	1.79
GO:0050994	regulation of lipid catabolic process	3	1.09	1.79
GO:0006957	complement activation, alternative pathway	2	1.51	1.77
GO:0097320	plasma membrane tubulation	2	1.51	1.77
GO:1903729	regulation of plasma membrane organization	2	1.51	1.77
GO:0048731	system development	32	0.21	1.76
GO:0050808	synapse organization	5	0.75	1.76
GO:0071900	regulation of protein serine/threonine kinase activity	8	0.54	1.74
GO:0032271	regulation of protein polymerization	5	0.74	1.74
GO:0010676	positive regulation of cellular carbohydrate metabolic process	3	1.07	1.73
GO:0010035	response to inorganic substance	8	0.54	1.73
GO:0034764	positive regulation of transmembrane transport	5	0.73	1.73
GO:0051046	regulation of secretion	10	0.46	1.73
GO:0070208	protein heterotrimerization	2	1.48	1.73
GO:0070374	positive regulation of ERK1 and ERK2 cascade	5	0.73	1.71
GO:0007169	transmembrane receptor protein tyrosine kinase signaling pathway	8	0.53	1.70
GO:0003012	muscle system process	6	0.64	1.68
GO:0046628	positive regulation of insulin receptor signaling pathway	2	1.45	1.68
GO:0052548	regulation of endopeptidase activity	7	0.57	1.68
GO:0060192	negative regulation of lipase activity	2	1.45	1.68
GO:0071801	regulation of podosome assembly	2	1.45	1.68
GO:0080182	histone H3-K4 trimethylation	2	1.45	1.68
GO:2001028	positive regulation of endothelial cell chemotaxis	2	1.45	1.68
GO:0017144	drug metabolic process	9	0.48	1.68
GO:0048732	gland development	7	0.57	1.68
GO:0051223	regulation of protein transport	9	0.48	1.68
GO:0031333	negative regulation of protein complex assembly	4	0.84	1.68

Term ID #	Term Description	Count	Enrichment	-Log10PV
GO:0032272	negative regulation of protein polymerization	3	1.04	1.68
GO:0010243	response to organonitrogen compound	11	0.42	1.67
GO:0000902	cell morphogenesis	9	0.48	1.67
GO:0007051	spindle organization	4	0.84	1.67
GO:0043280	positive regulation of cysteine-type endopeptidase activity involved in apoptotic process	4	0.84	1.67
GO:0048519	negative regulation of biological process	36	0.18	1.67
GO:0099175	regulation of postsynapse organization	3	1.03	1.67
GO:0001956	positive regulation of neurotransmitter secretion	2	1.42	1.66
GO:0032233	positive regulation of actin filament bundle assembly	3	1.02	1.65
GO:0051129	negative regulation of cellular component organization	9	0.48	1.65
GO:0071230	cellular response to amino acid stimulus	3	1.02	1.65
GO:0050806	positive regulation of synaptic transmission	4	0.82	1.65
GO:0009968	negative regulation of signal transduction	13	0.37	1.64
GO:0050789	regulation of biological process	66	0.1	1.63
GO:0034375	high-density lipoprotein particle remodeling	2	1.39	1.63
GO:0043691	reverse cholesterol transport	2	1.39	1.63
GO:0045725	positive regulation of glycogen biosynthetic process	2	1.39	1.63
GO:1903578	regulation of ATP metabolic process	3	1.01	1.63
GO:0060341	regulation of cellular localization	10	0.44	1.62
GO:0035296	regulation of tube diameter	4	0.81	1.62
GO:0051592	response to calcium ion	4	0.81	1.62
GO:0051707	response to other organism	13	0.37	1.61
GO:0120031	plasma membrane bounded cell projection assembly	7	0.55	1.61
GO:0035023	regulation of Rho protein signal transduction	4	0.81	1.60
GO:0001503	ossification	5	0.69	1.60
GO:0042423	catecholamine biosynthetic process	2	1.37	1.60
GO:1903421	regulation of synaptic vesicle recycling	2	1.37	1.60
GO:0031056	regulation of histone modification	4	0.8	1.58
GO:0032717	negative regulation of interleukin-8 production	2	1.35	1.56
GO:1900273	positive regulation of long-term synaptic potentiation	2	1.35	1.56
GO:0010941	regulation of cell death	16	0.31	1.55
GO:0097746	regulation of blood vessel diameter	4	0.79	1.55
GO:0051716	cellular response to stimulus	42	0.15	1.54
GO:0042981	regulation of apoptotic process	15	0.32	1.53
GO:0051004	regulation of lipoprotein lipase activity	2	1.32	1.53
GO:0070584	mitochondrion morphogenesis	2	1.32	1.53
GO:1900371	regulation of purine nucleotide biosynthetic process	3	0.96	1.53
GO:1902176	negative regulation of oxidative stress-induced intrinsic apoptotic signaling pathway	2	1.32	1.53
GO:1903530	regulation of secretion by cell	9	0.45	1.53
GO:0048856	anatomical structure development	36	0.17	1.53
GO:0030833	regulation of actin filament polymerization	4	0.78	1.52
GO:0051225	spindle assembly	3	0.96	1.52
GO:0034394	protein localization to cell surface	2	1.3	1.51
GO:0072359	circulatory system development	10	0.42	1.51
GO:0000122	negative regulation of transcription by RNA polymerase II	10	0.42	1.50
GO:0002757	immune response-activating signal transduction	6	0.58	1.50
GO:0022898	regulation of transmembrane transporter activity	5	0.66	1.50
GO:0031324	negative regulation of cellular metabolic process	21	0.25	1.50
GO:0043086	negative regulation of catalytic activity	10	0.42	1.50
GO:0062012	regulation of small molecule metabolic process	6	0.58	1.50
GO:0032535	regulation of cellular component size	6	0.58	1.50
GO:0003008	system process	17	0.29	1.50
GO:0051172	negative regulation of nitrogen compound metabolic process	20	0.26	1.49

Term ID #	Term Description	Count	Enrichment	-Log10PV
GO:0002526	acute inflammatory response	3	0.94	1.49
GO:0050995	negative regulation of lipid catabolic process	2	1.28	1.49
GO:0006952	defense response	13	0.35	1.48
GO:0071345	cellular response to cytokine stimulus	11	0.39	1.48
GO:0016310	phosphorylation	13	0.34	1.48
GO:0051241	negative regulation of multicellular organismal process	12	0.36	1.47
GO:0071902	positive regulation of protein serine/threonine kinase activity	6	0.57	1.47
GO:0030325	adrenal gland development	2	1.26	1.46
GO:0090140	regulation of mitochondrial fission	2	1.26	1.46
GO:0030705	cytoskeleton-dependent intracellular transport	4	0.75	1.45
GO:0010951	negative regulation of endopeptidase activity	5	0.64	1.45
GO:0033344	cholesterol efflux	2	1.24	1.43
GO:0051194	positive regulation of cofactor metabolic process	2	1.24	1.43
GO:0045861	negative regulation of proteolysis	6	0.56	1.42
GO:0009894	regulation of catabolic process	10	0.4	1.42
GO:0007265	Ras protein signal transduction	4	0.73	1.42
GO:1903827	regulation of cellular protein localization	7	0.5	1.41
GO:0019433	triglyceride catabolic process	2	1.23	1.40
GO:0050794	regulation of cellular process	62	0.09	1.40
GO:0030838	positive regulation of actin filament polymerization	3	0.89	1.40
GO:0048259	regulation of receptor-mediated endocytosis	3	0.89	1.40
GO:0030307	positive regulation of cell growth	4	0.72	1.39
GO:0032502	developmental process	37	0.16	1.39
GO:0045927	positive regulation of growth	5	0.62	1.39
GO:1901700	response to oxygen-containing compound	14	0.31	1.39
GO:0030041	actin filament polymerization	2	1.21	1.39
GO:0043534	blood vessel endothelial cell migration	2	1.21	1.39
GO:0051220	cytoplasmic sequestering of protein	2	1.21	1.39
GO:0032990	cell part morphogenesis	7	0.49	1.38
GO:0050807	regulation of synapse organization	4	0.72	1.38
GO:0001649	osteoblast differentiation	3	0.88	1.38
GO:0007568	aging	5	0.62	1.38
GO:0043066	negative regulation of apoptotic process	10	0.39	1.38
GO:0043255	regulation of carbohydrate biosynthetic process	3	0.88	1.38
GO:0045088	regulation of innate immune response	6	0.54	1.38
GO:0002697	regulation of immune effector process	6	0.54	1.38
GO:0007163	establishment or maintenance of cell polarity	4	0.71	1.38
GO:0031349	positive regulation of defense response	6	0.54	1.37
GO:0048167	regulation of synaptic plasticity	4	0.71	1.37
GO:0051354	negative regulation of oxidoreductase activity	2	1.19	1.37
GO:2001171	positive regulation of ATP biosynthetic process	2	1.19	1.37
GO:0006638	neutral lipid metabolic process	3	0.87	1.36
GO:0050821	protein stabilization	4	0.7	1.36
GO:0071417	cellular response to organonitrogen compound	7	0.48	1.36
GO:0090162	establishment of epithelial cell polarity	2	1.18	1.35
GO:0002758	innate immune response-activating signal transduction	4	0.7	1.35
GO:0031329	regulation of cellular catabolic process	9	0.41	1.34
GO:0007179	transforming growth factor beta receptor signaling pathway	3	0.86	1.34
GO:0008015	blood circulation	6	0.53	1.34
GO:0002684	positive regulation of immune system process	10	0.38	1.33
GO:0009892	negative regulation of metabolic process	22	0.22	1.33
GO:0045892	negative regulation of transcription, DNA-templated	12	0.33	1.33
GO:0060191	regulation of lipase activity	3	0.85	1.32
GO:0009888	tissue development	15	0.29	1.32

Term ID #	Term Description	Count	Enrichment	-Log10PV
GO:0045922	negative regulation of fatty acid metabolic process	2	1.15	1.31
GO:0044089	positive regulation of cellular component biogenesis	7	0.47	1.31
GO:0046890	regulation of lipid biosynthetic process	4	0.68	1.31
GO:0031327	negative regulation of cellular biosynthetic process	14	0.3	1.31
GO:0048666	neuron development	9	0.4	1.30
GO:0045055	regulated exocytosis	25	0.88	11.30
**Decreased in post LT fibrosis**				
GO:0002576	platelet degranulation	10	1.49	8.67
GO:0007010	cytoskeleton organization	16	0.83	6.24
GO:0016192	vesicle-mediated transport	20	0.67	6.14
GO:0045055	regulated exocytosis	13	0.88	5.38
GO:0030029	actin filament-based process	10	0.91	4.05
GO:0030036	actin cytoskeleton organization	9	0.93	3.77
GO:0016043	cellular component organization	29	0.35	3.66
GO:0022607	cellular component assembly	19	0.51	3.62
GO:0007229	integrin-mediated signaling pathway	5	1.38	3.48
GO:0097435	supramolecular fiber organization	8	0.92	3.28
GO:0006953	acute-phase response	4	1.55	3.22
GO:0006810	transport	24	0.37	2.92
GO:0035296	regulation of tube diameter	5	1.19	2.80
GO:0090066	regulation of anatomical structure size	8	0.84	2.80
GO:0042730	fibrinolysis	3	1.76	2.74
GO:0051179	localization	27	0.31	2.74
GO:0097746	regulation of blood vessel diameter	5	1.16	2.74
GO:0030193	regulation of blood coagulation	4	1.32	2.57
GO:0031589	cell-substrate adhesion	5	1.09	2.52
GO:0003012	muscle system process	6	0.92	2.37
GO:0006996	organelle organization	19	0.38	2.31
GO:0002376	immune system process	16	0.43	2.21
GO:0007015	actin filament organization	5	1	2.19
GO:2001237	negative regulation of extrinsic apoptotic signaling pathway	4	1.19	2.19
GO:0007044	cell-substrate junction assembly	3	1.48	2.17
GO:0043152	induction of bacterial agglutination	2	2.12	2.17
GO:0001775	cell activation	10	0.59	2.12
GO:0006952	defense response	11	0.55	2.12
GO:0007160	cell-matrix adhesion	4	1.13	2.05
GO:0006897	endocytosis	7	0.74	2.01
GO:0065003	protein-containing complex assembly	12	0.5	2.01
GO:0043933	protein-containing complex subunit organization	13	0.47	2.00
GO:0007167	enzyme linked receptor protein signaling pathway	8	0.66	2.00
GO:0008015	blood circulation	6	0.81	2.00
GO:0033622	integrin activation	2	1.95	2.00
GO:0006936	muscle contraction	5	0.91	1.97
GO:0034329	cell junction assembly	4	1.07	1.97
GO:0031532	actin cytoskeleton reorganization	3	1.32	1.92
GO:0031639	plasminogen activation	2	1.86	1.91
GO:0051640	organelle localization	7	0.69	1.84
GO:0034116	positive regulation of heterotypic cell-cell adhesion	2	1.79	1.81
GO:0051641	cellular localization	14	0.41	1.78
GO:0140056	organelle localization by membrane tethering	4	1	1.77
GO:0007017	microtubule-based process	7	0.66	1.76
GO:0098657	import into cell	7	0.66	1.75
GO:1903524	positive regulation of blood circulation	3	1.23	1.75
GO:0030198	extracellular matrix organization	5	0.83	1.71
GO:0048268	clathrin coat assembly	2	1.67	1.67
GO:0001568	blood vessel development	6	0.71	1.65
GO:0008217	regulation of blood pressure	4	0.96	1.65

Term ID #	Term Description	Count	Enrichment	-Log10PV
GO:0002064	epithelial cell development	4	0.95	1.65
GO:0050878	regulation of body fluid levels	6	0.7	1.60
GO:0051258	protein polymerization	3	1.16	1.60
GO:0045087	innate immune response	7	0.62	1.56
GO:0003008	system process	12	0.42	1.56
GO:0032970	regulation of actin filament-based process	5	0.77	1.55
GO:0045185	maintenance of protein location	3	1.1	1.47
GO:0031032	actomyosin structure organization	3	1.09	1.47
GO:0070527	platelet aggregation	2	1.5	1.46
GO:0022407	regulation of cell-cell adhesion	5	0.74	1.43
GO:0030194	positive regulation of blood coagulation	2	1.49	1.43
GO:0032967	positive regulation of collagen biosynthetic process	2	1.49	1.43
GO:0007166	cell surface receptor signaling pathway	13	0.37	1.43
GO:0072378	blood coagulation, fibrin clot formation	2	1.47	1.43
GO:2000352	negative regulation of endothelial cell apoptotic process	2	1.46	1.42
GO:0045595	regulation of cell differentiation	11	0.41	1.39
GO:0030866	cortical actin cytoskeleton organization	2	1.43	1.37
GO:0110053	regulation of actin filament organization	4	0.83	1.36
GO:0006911	phagocytosis, engulfment	2	1.41	1.35
GO:1900026	positive regulation of substrate adhesion-dependent cell spreading	2	1.4	1.33
GO:0045907	positive regulation of vasoconstriction	2	1.38	1.31

Supplemental Table 6. Feeding, growth and lipid accumulation caused by HFD in C57Bl6/J and AJ mice.

	LFD		*HFD*	
	C57Bl6/J	AJ	C57Bl6/J	AJ
Food consumption (g/d)	2.7±0.1	2.6±0.1	2.7±0.1	2.5±0.2
BW gain (g/wk)	0.4±0.1	0.6±0.1	1.5±0.1^{a}	0.6±0.1^{b}
LW (% of BW)	3.5±0.1	4.1±0.1	6.5±0.4^{a}	4.6±0.1^{b}
Hepatic TG (µg/mg liver)	5.3±2.1	5.4±1.3	140±41^{a}	17.6±5.5^{b}
Hepatic FFA (ng/mg liver)	0.1±0.1	0.2±0.1	7.5±1.9^{a}	1.7±1.0^{b}
Hepatic TC (µg/mg liver)	0.4±0.1	0.9±0.1	7.0±1.6^{a}	5.4±1.5^{a}

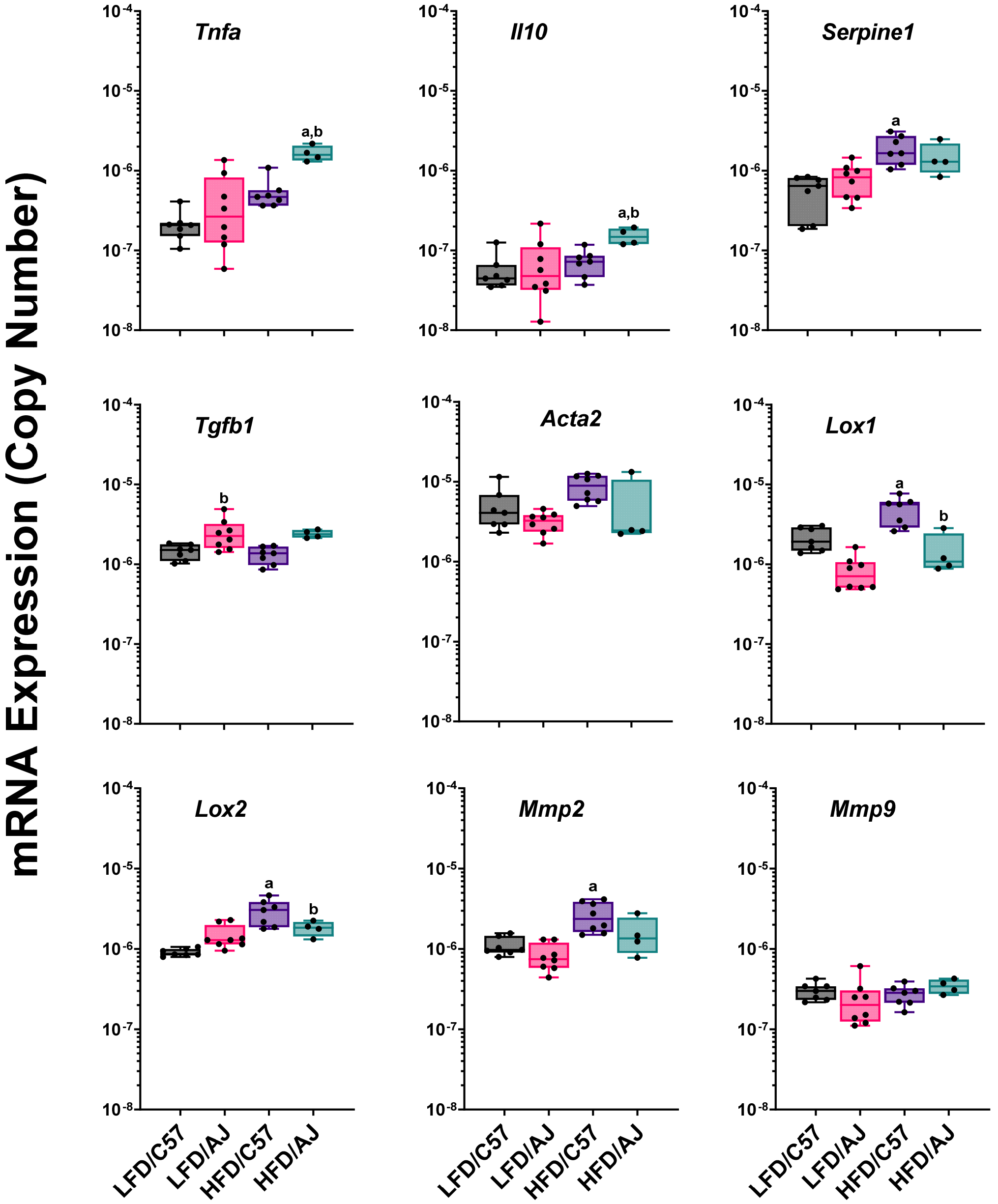

Supplemental Figure 1-Li et al.
